# Endogenous Mobilization of Mesenchymal Stromal Cells: A Pathway for Interorgan Communication?

**DOI:** 10.3389/fcell.2020.598520

**Published:** 2021-01-08

**Authors:** Amandine Girousse, Maxime Mathieu, Quentin Sastourné-Arrey, Sylvie Monferran, Louis Casteilla, Coralie Sengenès

**Affiliations:** ^1^Stromalab, Université de Toulouse, CNRS ERL5311, EFS, INP-ENVT, INSERM U1031, Université Paul Sabatier, Toulouse, France; ^2^Sprott Center for Stem Cell Research, Ottawa Hospital Research Institute, Department of Cellular and Molecular Medicine, University of Ottawa, Ottawa, ON, Canada

**Keywords:** adipose tissue, native mesenchymal stromal cell, stroma homeostasis, endogenous reservoir, rare cells in circulation

## Abstract

To coordinate specialized organs, inter-tissue communication appeared during evolution. Consequently, individual organs communicate their states *via* a vast interorgan communication network (ICN) made up of peptides, proteins, and metabolites that act between organs to coordinate cellular processes under homeostasis and stress. However, the nature of the interorgan signaling could be even more complex and involve mobilization mechanisms of unconventional cells that are still poorly described. Mesenchymal stem/stromal cells (MSCs) virtually reside in all tissues, though the biggest reservoir discovered so far is adipose tissue where they are named adipose stromal cells (ASCs). MSCs are thought to participate in tissue maintenance and repair since the administration of exogenous MSCs is well known to exert beneficial effects under several pathological conditions. However, the role of endogenous MSCs is barely understood. Though largely debated, the presence of circulating endogenous MSCs has been reported in multiple pathophysiological conditions, but the significance of such cell circulation is not known and therapeutically untapped. In this review, we discuss current knowledge on the circulation of native MSCs, and we highlight recent findings describing MSCs as putative key components of the ICN.

## Introduction

Each organ is a combination of a functional compartment, the parenchyma, and a stromal compartment, the stroma, supporting the parenchymal cells of the organ (Feeback, 1987). The main function of the stromal compartment is to structure and remodel functional tissue in order to ensure organ homeostasis (Scadden, 2012). In normal tissue, stroma maintains the tissue microenvironment and sustains cell growth in various ways with spatial and temporal self-limitations (Huet et al., 2019). Conversely, stroma imbalance nurtures organ imbalance, which can eventually lead to tumor progression (Valkenburg et al., 2018). Among the cell types residing in the stroma, mesenchymal stem/stromal cells (MSCs) are key components allowing stroma’s supportive function. MSCs attract lots of attention because they hold great promise for a multitude of emerging therapies in regenerative medicine since they promote tissue repair in various degenerative contexts such as osteoarthritis, bone defects, myocardial infarction, inflammatory bowel disease, or neurodegenerative disorders. As such, they have been the subject of clinical trials for more than 20 years (Galipeau and Sensébé, 2018; Pittenger et al., 2019). MSC identification and characterization rely on *in vitro* work, and long steps of culture are needed to collect a usable amount of cells (Dominici et al., 2006). Culture-expanded MSCs consist of a heterogeneous population of cells exhibiting various phenotypes and functional properties, and the extent of these properties depends on the tissue, donor, and species of origin, isolation technique, and culturing protocols (Ankrum et al., 2014). Such variations are known to limit the potential of MSCs for clinical translation, and strategies to enhance engraftment are needed (Hou et al., 2005; Hénon, 2020).

In the past few years, investigating the endogenous repair mechanisms of injured tissues has paved the way for future “*in situ*” strategies to potentiate the body’s own repair capacity (Andreas et al., 2014). In this regard, pharmacological activation of endogenous stem cell mobilization from either the blood or a tissue-specific niche is a promising approach (Krankel et al., 2011). Consequently, both triggering and controlling the endogenous mobilization of MSCs represent an additional strategy to achieve effective tissue repair and regeneration. In this review, we present the current state of knowledge and unresolved gaps about the circulation of endogenous MSCs and propose MSC interorgan trafficking as a complementary pathway of communication.

## What Do We Know About the Circulation of Endogenous Mesenchymal Stem/Stromal Cells?

### Circulating Mesenchymal Stem/Stromal Cells: Myth or Reality?

Studies reporting the mobilization, circulation, and recruitment of endogenous MSCs are sparse and heterogeneous (Roufosse et al., 2004) and generated lots of conflicting results (Ojeda-Uribe et al., 1993; Lazarus et al., 1997; Zvaifler et al., 2000; Wexler et al., 2003). Consequently, the presence of blood circulating MSCs is still debated (Mansilla et al., 2006; Wang et al., 2006; Hoogduijn et al., 2014).

Yet, several studies show that endogenous MSCs are found in the bloodstream of various species, but their frequency is rare [0–0.025 colonies/10^*e*^6 of peripheral blood mononuclear cells (He et al., 2007)]. Conversely, the circulation of endogenous MSCs greatly increases in response to various types of injuries. Indeed, skeletal traumas, regardless of their severity (Alm et al., 2010), cardiomyopathies (Marketou et al., 2014, 2015), coronary syndrome (Wojakowski et al., 2008), skin burns (Mansilla et al., 2006), liver damages (Chen et al., 2010; Liu et al., 2015), and some types of cancers (Fernandez et al., 1997; Bian et al., 2009) are some examples of clinical situations triggering this increase. Whether endogenous MSCs circulate *in vivo* is not a matter of debate anymore but rather a matter of methods of investigation, time frame (Churchman et al., 2020), and clinical context. Such limitations relate to a lack of precise knowledge of functional, phenotypic, and molecular criteria that define endogenous circulating MSCs.

### Immunophenotypic Characteristics of Circulating Endogenous Mesenchymal Stem/Stromal Cells

**FIGURE 1:**
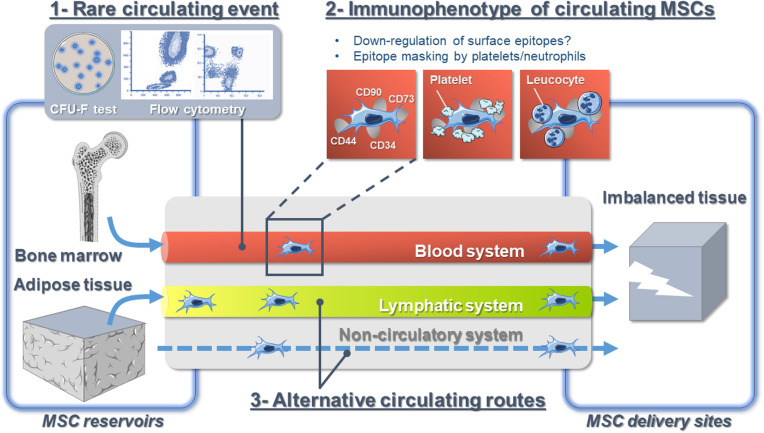
The hypotheses explaining the controversy over endogenous mesenchymal stromal cell circulation *in vivo*.

Despite extensive efforts to characterize MSCs, the definition of *in vivo* identity(ies) of MSCs is still very obscure (Parekkadan and Milwid, 2010). In humans, the canonical MSC surface marker combination CD13^+^/CD44^+^/CD73^+^/CD90^+^/CD105^+^/CD34^–^/CD31^–^/CD45^–^ directly derives from their *in vitro* culture expansion (Dominici et al., 2006). However, many factors, from the harvesting methodology to the conditions of cell culture, dramatically influence MSC phenotype and functions (Bara et al., 2014; Jones and Schäfer, 2015; Pittenger et al., 2019; Walter et al., 2020). In that regard, we and others have demonstrated that cell surface marker profiles of *in vitro* expanded human MSCs differ compared to freshly isolated cells and those residing in their native microenvironment (Sengenès et al., 2005; Maumus et al., 2011; Bara et al., 2014). In particular, the absence of CD34 is considered among the prerequisites to identify MSCs; however, we have shown that CD34 is strongly expressed in native adipose-derived MSCs and that cell culture abolishes its expression (Sengenès et al., 2005; Maumus et al., 2011). Moreover, though some of the MSC markers appear constitutively expressed regardless of environment (Jones et al., 2006), “immunophenotypic drifts” are expected while MSCs circulate. Indeed, the expression of membrane markers such as CD29, CD44, CD73, and CD90, which all regulate MSC adhesion/migration processes, is known to change dramatically to allow MSC detachment and further migration (Rege and Hagood, 2006; Ode et al., 2011; Qian et al., 2012; Xu and Li, 2014). Consequently, using flow cytometry analysis with a combination of surface markers (validated *in vitro*) to detect circulating native MSCs may lead to underestimation and generates conflicting results when compared with studies using functional assays to detect MSCs [such as colony-forming unit-fibroblast (CFU-F) activity] (Fellous et al., 2020; Figure 1). Indeed, the level of blood circulating CD45^–^/CD271^+^ MSCs shows higher correlation to CFU-F numbers than the one of CD45^–^/CD73^+^/CD90^+^/CD105^+^ MSCs (Rebolj et al., 2018). This illustrates that understanding MSC heterogeneity holds promise for refining the definition of MSCs. In that regard, the analysis of MSC heterogeneity from various tissue [bone marrow (BM), adipose tissue (AT), skeletal muscle] is under active investigation using single-cell RNA sequencing technologies (Burl et al., 2018; Hepler et al., 2018; Baryawno et al., 2019; Wolock et al., 2019). However, though powerful, those studies will inform about the signature(s) of native tissue-resident MSC subpopulations but will fail for circulating MSCs. Interestingly, high-throughput technology capable of efficiently capturing without marker-based approach and molecularly interrogating rare cells in the circulation at single-cell resolution is under development to study circulating tumor cells (CTCs) (Cheng et al., 2008). Those technologies will be of great utility both to capture and to enable single-cell transcriptome analysis of rare and limited cell populations of circulating endogenous MSCs.

### How Do Endogenous Mesenchymal Stem/Stromal Cells Navigate in the Bloodstream?

Little is known about the behavior of MSCs in flowing blood, and our current understanding mostly derives from intravascular infused cultured-expanded MSCs from which we could infer the behavior of native MSCs. While circulating MSCs are always considered to be isolated cells floating in the bloodstream, recent studies demonstrated the close interaction of MSCs with the blood microenvironment. Indeed, using *in vivo* confocal microscopy, it has been reported that the majority of intravascular MSCs are in contact with platelets and/or neutrophils (Teo et al., 2015). Additionally, BM-derived MSCs bind platelets that shield them from surface adhesion, so that they barely adhere at all in the blood flow *via* a mechanism involving podoplanin, the endogenous ligand for C-type lectin-like receptor 2 (CLEC-2) (Sheriff et al., 2018; Ward et al., 2019). CLEC-2 is being expressed broadly, including in platelets, inflammatory leukocytes, and lymphatic endothelial cells. Moreover, platelet depletion decreases MSC trafficking to sites of injury (Langer et al., 2009; Teo et al., 2015). Platelet functions extend beyond the immediate environment of the thrombus (Golebiewska and Poole, 2015). For instance, they play important roles for tissue regeneration (Eisinger et al., 2018), and they also contribute to tumor metastasis (Tesfamariam, 2016). Indeed, it is admitted that CTCs are partly covered with platelets to provide them with “stealth” properties and help their survival in the circulation, where they are challenged by physical forces in the circulation (Nieswandt et al., 1999; Heeke et al., 2019). Whether circulating endogenous MSCs are not single cells traveling the blood alone but are accompanied by other cell types and partners possibly modifying their immunophenotype needs more investigations (Figure 1). However, targeting the interaction of MSCs with other cells is a promising tool and future research to improve endogenous MSC detection, collection, and trafficking.

#### What if Not Just the Blood?

The peripheral blood is considered as the *bona fide* route for native MSC trafficking (He et al., 2007). Indeed, the detection of blood-borne CFU-Fs was earlier (Maximow, 1928) than the detection of BM CFU-Fs (Friedenstein et al., 1968, 1970). However, it is well established that some types of stem cells such as hematopoietic stem cells recirculate daily between the BM and the blood and egress to extramedullary tissues *via* the lymphatic system (Massberg et al., 2007). Until a few years ago, the composition of the lymphatic fluid was virtually unknown. This lack of knowledge was mostly due to the technical difficulty in cannulating lymphatic vessels and the small amount of collected fluid. Over time, some of these technical issues have been resolved, and as such, lymph “omic” composition in physiological and pathological conditions received a lot of attention (Santambrogio, 2018). However, the precise cellular composition of lymph is still obscure, and immune cell transit was mostly investigated (Platt and Randolph, 2013). Yet, we have demonstrated that MSCs originating from AT, the ASCs (Zuk et al., 2001; Gimble and Guilak, 2003), are found in the lymph fluid in response to lymph node inflammation (Gil-Ortega et al., 2013). Other studies indicate that systemically infused MSCs can be found in secondary lymphoid organs [e.g., mesenteric lymph nodes after intracardiac infusion (Li et al., 2012), lymph nodes, Peyer patches, spleen (Schwarz et al., 2014)]. Interestingly, Han et al. (2020) very recently reported the presence of lots of circulating cells able to form spheroids in the thoracic duct of a mouse model of melanoma, though distant metastases were not developed. Altogether, those data suggest that as cancer cells do, MSCs may exploit several bodily fluid systems as natural transportation routes (Follain et al., 2020; Figure 1).

Whether the clinical context, fluid biomechanics, and tissue microenvironment have a role in the initial choice of the fluid route is unknown. As well, accessibility of blood and lymphatic vasculature may strongly influence the pathway taken for MSCs to transit. Finally, flow velocities and shear stress are lower in lymphatic vessels (Dixon et al., 2006), and lymphatic dissemination has been suggested as less deleterious than dissemination through the blood for some type of cancer cells (Wong and Hynes, 2006). Lymph fluid could thus represent a more favorable route for MSCs since their survival may benefit from the passive, low-shear system of fluid transport characteristic of lymphatics. Consequently, an improved understanding of this process might provide a new avenue for targeting MSC transit and might explain conflicting results. At last, the fibroblastic nature of MSCs allows considering extra-circulatory alternative routes, such as connective tissues, for MSC mobilization (Figure 1). The potential for such trafficking events, putative mechanisms, and potential functional roles represents important questions for future investigation.

## Which Physiological Reservoirs may be Mobilized?

MSCs reside in virtually all postnatal organs and tissues; however, not all organs contain the same amount of MSCs (da Silva Meirelles et al., 2006; Crisan et al., 2009). BM is generally considered as the major reservoir of mobilizable MSCs (Koh et al., 2007; Koning et al., 2013). Nevertheless, together with the absence of unique specific markers, the lack of MSC tissue-specific markers impairs the parallel analysis of various physiological reservoirs. Consequently, it is very likely that the role played by extramedullary organs in participating in the pool of circulating endogenous MSCs is underestimated. Indeed, AT is a large source of MSCs, named ASCs (Zuk et al., 2001; Gimble and Guilak, 2003). The uncultured stroma–vascular fraction (SVF) from AT usually contains up to 30% of ASCs. This is 2,500-fold more than the frequency found in BM (Fraser et al., 2008; Baer and Geiger, 2012). Consequently, AT represents so far the largest physiological reservoir of MSCs.

In the attempt of investigating to what extent AT contributes to the pool of circulating endogenous MSCs, we and others have shown that endogenous ASCs are mobilizable and that such mobilization is triggered in response to various types of stresses from inflammation to fat overload (Zhang et al., 2009; Kolonin, 2012; Gil-Ortega et al., 2013, 2014; Girousse et al., 2019). Consequently, AT also largely accounts for the pool of circulating endogenous MSCs, but animal models are still needed to clearly evaluate the respective part played by BM vs. AT.

## Why Do Endogenous Mesenchymal Stem/Stromal Cells Circulate?

Whatever the reservoir, circulating endogenous MSCs belong to the group of blood-circulating rare cell populations, classified by Schreier and Triampo (2020) into “constructive” and “destructive” cell types. MSCs are mostly considered as constructive cell types because of their repair and/or homeostasis maintenance properties. The current knowledge on the functional roles of MSCs mainly relies on studies using *in vitro*-expanded MSCs (Keating, 2012; Galipeau and Sensébé, 2018; Pittenger et al., 2019). The struggles in clearly defining native MSCs negatively influence advancement in understanding their role(s) *in vivo* and what is more the role of their circulation. Last, since MSCs virtually reside in all postnatal organs and tissues (da Silva Meirelles et al., 2006; Crisan et al., 2009), one may wonder why MSCs circulate toward distant “injured/inflamed” sites, while resident ones could perform the same activities.

### The Interorgan Communication Network

**FIGURE 2:**
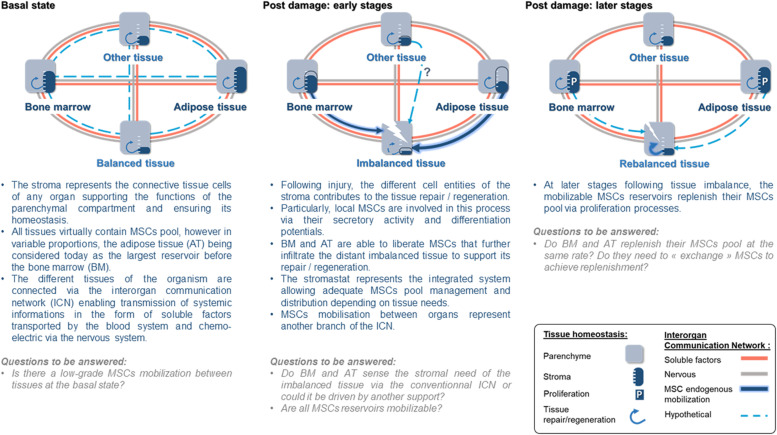
The stromastat hypothesis within the interorgan communication network during basal and after tissue damage.

The long-term maintenance of an organism’s homeostasis and health relies on the accurate regulation of organ–organ communication (Silverthon et al., 2009). To do so, the central nervous system regulates many organ behaviors using hormones or neurons and organs developed systems to directly communicate their states to one another. This interorgan communication network (ICN) is made up of soluble factors such as peptides, proteins, and metabolites that act between organs to coordinate essential and specialized cellular processes under homeostasis and stress (Droujinine and Perrimon, 2016; Figure 2). Recent studies show that more than 15% of the protein-coding genome encodes for roughly 3,000 secreted proteins, but only a handful of them has been properly annotated (Uhlen et al., 2010; Lindskog, 2015). Consequently, the nature of the ICN remains largely a mystery (Droujinine and Perrimon, 2013). The interorgan communication is seen to occur through secreted molecules; however, accumulating data show that organs communicate their state *via* other ways. For instance, extracellular vesicles (EVs) have emerged as a novel messaging system of the organism, mediating cell–cell and interorgan communication (Gould et al., 2003). EVs are secreted membranous structures, entrapping nucleic acids, diverse cellular proteins, and metabolites, and are predicted to transfer their packaged molecules from one cell to another (Gould et al., 2003). EVs traffic to local or distant targets to execute defined biological functions (Théry et al., 2009; Thomou et al., 2017; Margolis and Sadovsky, 2019). Consequently, the ICN encompasses other modes of communication than secreted molecules, and as such, whether the circulation of MSCs is a way of communication between organs needs to be considered.

### Circulating Mesenchymal Stem/Stromal Cells, a Way to Communicate Between Bodily Stromas

As stated above, the stromal compartment of each organ structures, nurtures, and remodels the functional compartment to ensure organ homeostasis. MSCs, being a central cellular component of bodily stromas, can be viewed as stroma “sentinel,” sensing stromal state and ultimately the organ state. The following scenario regarding the role of circulating endogenous MSCs may be proposed.

Just as suggested for the regulation of body temperature or AT mass in the form of thermostat and adipostat, respectively, bodily stromas could be regulated by a set point to ensure the organism’s homeostasis that we will name here the “stromastat.” Organ failure leading to stromastat modification could be detected by resident MSCs and trigger the early and rapid transfer from MSC reservoirs to the failing organ. This early transfer of MSCs would support the resident pool of MSCs to allow the rebalance of the failing organ. In parallel, to ensure stromastat, data report that the mobilized reservoir may be replenished (Koning et al., 2013; Figure 2). Thus, a possible answer to the meaning of MSC circulation could be that MSCs may represent the cellular part of the ICN. Indeed, evidence that MSCs may belong to the ICN is accumulating. For instance, following organ imbalance (e.g., inflammation, metabolic stress), we observed that ASCs transfer very early from AT toward inflamed lymph nodes (Gil-Ortega et al., 2013) or injured/remodeling skeletal muscle (Girousse et al., 2019). Similar results were also reported for BM-derived MSCs in response to other inflammatory/injury contexts such as myocardial infarction (Fukuda and Fujita, 2005), cranio cerebral trauma (Deng et al., 2011), and encephalomyelitis (Koning et al., 2013). Interestingly, independently of the clinical context or the reservoir investigated, the common point of those studies is the kinetic with which MSCs transfer from their reservoir to the unbalanced site. Such interorgan MSC transfer involves few amounts of cells when compared to the pool of local MSCs. However, we and others have demonstrated that, though discrete, such infiltration dramatically impacts the fate of the organ repair/regeneration/remodeling (Kumar and Ponnazhagan, 2012; Hu et al., 2013; Koning et al., 2013; Girousse et al., 2019). In addition to this, the impact of this rare MSC population could be amplified by the production of EVs, like an inverted funnel effect.

## Conclusion and Perspectives

Both the mobilization and circulation of endogenous MSCs in physiology and pathology are undoubtful as seen in the present review. However, there are still several questions to be resolved before understanding the meaning of such circulation. One can argue that this is merely explainable because of current available technologies and lack of MSC-specific markers. Indeed, being a population of rare cells in the blood, we have only scratched the surface of the potential of circulating MSCs in diagnostics and regenerative medicine. It appears that in case of “emergency,” MSCs traffic from adipose or BM reservoir toward distant “injured/inflamed/imbalanced” organs where infiltrated MSCs trigger local mechanisms to allow repair/regeneration. In return, the MSC provider replenishes its own reservoir so that both compartments balance their respective MSC pools, suggesting the presence of a set point that we suggested to name the “stromastat” (Figure 2). How the stromastat regulates organ responses to various stresses and pathological contexts is completely unexplored. This interorgan way of communication may be an unsuspected source of therapeutic targets to help in maintaining whole-organism homeostasis. At last, a better understanding of the control of endogenous MSC circulation, including the description of mobilization and attraction mechanisms, will represent an essential step that will condition their therapeutic potential.

## Author Contributions

All authors contributed to the design and writing of the manuscript. AG, MM, QS-A, SM, and LC proofread and given comments as well as suggestions. AG designed all the figures. CS supervised and finalized the manuscript.

## Conflict of Interest

The authors declare that the research was conducted in the absence of any commercial or financial relationships that could be construed as a potential conflict of interest.
